# Factors Influencing Utilization of HIV Testing Services among Boda-Boda Riders in Kabarole District, Southwestern Uganda: A Cross-Sectional Study

**DOI:** 10.1155/2021/8877402

**Published:** 2021-04-02

**Authors:** Vicent Ssekankya, Stanley Kamau Githaiga, Timothy Aleko, Esther Faith Munguciada, Vivian Patience Nabakka, Jolly Justine Kyalisiima, Alex Ndyabakira, Richard Migisha

**Affiliations:** ^1^Mbarara University of Science and Technology, P.O. Box 1410, Mbarara, Uganda; ^2^Infectious Diseases Research Collaboration, 2C Nakasero Hill Road. PO BOX 7475 Kampala, Uganda

## Abstract

**Background:**

HIV testing is an important step for entry and linkage into HIV care. Utilization of HIV testing services among transport workers may be challenging, because of the mobile nature of their jobs. We assessed utilization of HIV testing services and identified factors influencing the utilization of HIV testing services among motorcycle taxi (boda-boda) riders in Fort Portal Municipality, Kabarole District, Southwestern Uganda.

**Methods:**

We conducted a cross-sectional study among boda-boda riders, aged 18 years and above, from July 15 to July 29, 2020. We recruited participants through simple random sampling. Data were captured using a self-administered questionnaire. Binary logistic regression was used to identify factors associated with utilization of HIV testing services.

**Results:**

Of the 315 participants who received questionnaires, 305 (97%) responded. The mean age of the participants was 32 (±7.1) years and ranged from 18 to 55 years. Of the 305 participants, 238 reported having taken an HIV test and received results in the past 12 months, for an HIV testing utilization rate of 78.0% (95% CI: 73.0–82.6%). In multivariable analysis, participants who were less likely to utilize HIV testing services were those aged ≥30 years (aOR = 0.33; 95% CI: 0.16–0.70, *P* = 0.004) and those who had HIV-related stigma (aOR = 0.27; 95% CI: 0.08–0.88, *P* = 0.030). Participants who were more likely to utilize HIV testing services were those who knew HIV status of their primary partners (aOR = 4.23; 95% CI: 1.24–14.49, *P* = 0.022) and those who had good knowledge on HIV/AIDS (aOR = 3.94; 95% CI: 1.65–9.41, *P* = 0.002).

**Conclusions:**

Utilization of HIV testing services among the boda-boda riders in Fort Portal Municipality, Southwestern Uganda, was high. More efforts should focus on targeting older boda-boda riders for HIV testing, reduction of HIV-related stigma, improving knowledge on HIV/AIDS, and encouraging communication and disclosure between partners, in order to consolidate the gains made in HIV testing services in this bridge population.

## 1. Introduction

Globally, the number of individuals with HIV infection was estimated at 38 million in 2018 [1, 2]. Sub-Saharan Africa accounts for the majority (61%) of all HIV infections worldwide [1]. Recent data on HIV estimates in 2019 suggest that no country or region had met the 2020 target of 75% reduction in new HIV infections or HIV/AIDS-related deaths from 2010 indicators [2]. The Joint United Nations Programme on HIV/AIDS (UNAIDS) and other partners launched the 90–90–90 strategy in 2014, with the aim of ending the HIV pandemic by 2030: by the year 2020, 90% of persons living with HIV (PLHIV) should know their HIV status; 90% of all PLHIV should be on antiretroviral therapy; and 90% of individuals on antiretroviral treatment (ART) should have suppressed viral load [3]. With regard to the 90–90–90 targets, Uganda had registered good progress as of 2019; 90% of the PLHIV knew their HIV status, 96% of individuals who tested positive were on treatment, and of these, 87% had achieved viral suppression [4]. Therefore, HIV testing is of great public health importance, as there is evidence to suggest that more than half of the new HIV infections are caused by persons who are not aware of their HIV status [3]. Moreover, HIV counselling and testing are an important step for entry and linkage into HIV care/treatment.

“Boda-Boda” is a widely used term in Uganda, to refer to motorcycle taxis. In Uganda, the motorcycle taxi (boda-boda) is one of the fastest-growing businesses in the transport sector. This industry is a significant source of income, employing a large number of male youths in Uganda, and has contributed considerably to their social and economic well-being [5]. Over the years, transport workers, including long-distance truck drivers, taxi drivers, and boda-boda riders, have been identified as populations of interest [6]. Surveys conducted among boda-boda riders in central and western Uganda reported a higher HIV prevalence among the riders compared to their male counterparts [6, 7]. Furthermore, a lower HIV testing utilization rate of 37% [6] was previously reported among the boda-boda riders in Kampala than the rate (58%) reported among their male counterparts [8]. Other available data from across the globe also highlight high prevalence of risky sexual behaviors, such as having multiple sexual partners and unprotected sex in Cambodia [9] and Nigeria [10–12], among these mobile populations.

Utilization of HIV testing services among transport workers may be challenging, because of the nature of their jobs, which do not allow them time to go for the HIV testing services.

There are limited data on utilization of HIV testing services by the boda-boda riders in southwestern Uganda. In this cross-sectional survey, we aimed to determine the coverage of HIV testing services and identify factors associated with utilization of HIV testing services, among boda-boda riders in Kabarole District, in southwestern Uganda. Kabarole district is one of the districts in Uganda with the highest burden of HIV infections, with HIV prevalence rate of 16% [13]. Comparatively, the prevalence of HIV among adults in the 15–49-year age group in Uganda was estimated at 5.8% in 2018 [14].

## 2. Methods

### 2.1. Study Setting and Population

This was a cross-sectional study carried out from July 15 to July 29, 2020 in Fort Portal municipality, Kabarole District, southwestern Uganda. The district is part of the Kingdom of Tooro whose main town is Fort Portal. It lies approximately 320 kilometers, by road, west of Kampala, Uganda's capital. The district has an estimated population of 325,261 persons and 50% of these are male [15]. Kabarole district was purposively chosen because of the high prevalence of HIV in the district [13].

Our study participants were boda-boda riders aged 18 years and above, who had spent at least one year in this business, working within Fort Portal Municipality. The participants were recruited through simple random sampling. Briefly, a list and contacts of all the eligible participants were obtained from the chairperson of Boda-boda Riders' Association. Participants were then randomly selected and contacted through telephone calls. After explaining the study objectives, the participants were then invited to participate in the study. In the circumstance that there was no response, the call was repeated twice a day, for up to two consecutive days. If appropriate, a message (including a telephone number) was left and the participant was requested to contact the researcher if interested. In the event that a participant did not agree to participate or if he did not respond to the calls, he was replaced with yet another participant matching the inclusion requirements using the same procedure. All participants answered the questionnaire in a private setting at their respective boda-boda stages in the study area. If a participant was illiterate or semiliterate, the questionnaire was completed through an interview, whereby the researcher would read the questions to each participant and then record his answers in the questionnaire.

### 2.2. Data Collection Procedures

Data were collected through a pretested self-administered structured questionnaire. The questionnaire was prepared in English and then translated to the local language (Rutooro). A questionnaire, with both English and Rutoro questions/responses, was used during data collection.

The questionnaire captured sociodemographic characteristics (age, weekly income, religion, marital status, and duration in boda-boda business), health and sexual behavior characteristics (history of smoking, alcohol consumption, perceived risk of HIV infection, whether participant ever visited a health facility, and reasons for visiting health facility), HIV-related stigma, and knowledge on HIV/AIDs.

We assessed HIV knowledge using five questions: (1) whether a healthy-looking person can have the HIV virus, (2) whether HIV can be transmitted by sharing food, (3) whether HIV can be transmitted by mosquito bites, (4) whether HIV can be prevented through being faithful to one uninfected partner, and (5) whether HIV can be prevented through condom use. We categorized participants as having good HIV knowledge if they answered correctly >2 of the questions.

We assessed HIV-related stigma using four questions: (1) whether participant would be willing to care for a relative who becomes ill with HIV, (2) whether a participant would be willing to buy vegetables/fruits from a food seller who has the HIV virus, (3) whether a participant would allow his child to play with a child who has HIV virus, and (4) whether a participant would keep secret a family member with HIV infection. Participants who scored 2-4 were categorized as having stigma, whereas those who scored 0-1 were considered not to have HIV-related stigma.

Our dependent variable was utilization of HIV testing services. Participants were considered to have utilized HIV testing services if they had undergone an HIV test and received results to that test in the past 12 months.

### 2.3. Sample Size

The number of study participants required for the study was determined using the single population proportion formula in consideration of 95% confidence level, 5% precision, and design effect of 1 and 54% utilization rate for HIV testing, among residents of Kabarole District [16], from a source population of 1,000 boda-boda riders. The calculated final sample size was 305 participants, after inflation for a 10% nonresponse rate, using Epi Info (version 7.1.4.0, CDC, Atlanta US) statistical calculator.

### 2.4. Data Management and Analysis

Data were entered in EpiData3.1 software (EpiData, Odense, Denmark), then exported to STATA version 13 (StataCorp, College Station, Texas, USA) for analysis. We first determined the coverage of HIV testing services as a proportion of the participants who had an HIV test and received the results in the last 12 months, expressed as a percentage. Next, we compared participants' characteristics by HIV testing utilization status by using *χ*^2^ and Fischer's exact test (for categorical variables) and *t* tests (for continuous variables). Associations were quantified with simple and multivariable logistic regressions, reporting odds ratios (OR) and their corresponding 95% confidence intervals (CI) as our measures of association. Variables with *P* < 0.2 at univariable logistic regression were entered into multivariable model through stepwise backward elimination.

### 2.5. Ethical Considerations

The approval to conduct the study was obtained from Mbarara University of Science and Technology Research and Ethics Committee (MUST-REC) and assigned study number 08/01-20. Permission to conduct the study in the district was also obtained from district authorities and chairperson of Boda-boda Riders Association.

Written informed consent was obtained from all the participants, prior to participation. To ensure confidentiality, no individual identifiers such as names were used. The collected data were stored on a password-protected computer and kept in a lockable cupboard with restricted access.

## 3. Results

Of the 315 participants who received questionnaires, 305 filled the questionnaires, giving a response rate of 97%.

### 3.1. Demographic Characteristics

The demographic characteristics of the study participants are shown in Table 1. The mean age of the participants was 32 (±7.1) years and ranged from 18 to 55 years. All the participants were male. Of the 305 participants, most (61.6%) had attained primary education, 214 (70.2%) earned between 50,000 and 100,000 Ugandan shillings weekly, and 137 (44.9%) had spent 5–10 years in boda-boda business. Most (78%) of the participants were married and living with their partners. The majority (90.5%) of the study participants had at least a child. The distribution of age groups (*P* = 0.001) and duration in occupation (*P* = 0.039) was significantly different between participants who tested for HIV in the past 12 months and those who did not test for HIV (Table 1).

### 3.2. Behavioral, Health, and Social Characteristics

As shown in Table 2, nearly all (99%) of the study participants had ever visited a health facility. Of the 305 total participants, 19 (6.2%) reported history of smoking, 131 (43.0%) had a history of consuming alcohol in the past 12 months, and 273 (89.5%) were aware of the HIV status of their primary partners. With regard to the perceived risk for HIV infection in the next 12 months, most reported low risk perception (38.4%) or no risk at all (36.4%). The majority (90.8%) of the participants had good knowledge regarding HIV/AIDS. HIV-related stigma was low (4.6%) among the study participants (Table 2).

### 3.3. HIV Testing Services

**Figure 1:**
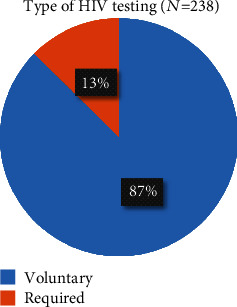
Types of HIV testing undertaken by boda-boda riders in Fort Portal Municipality, Kabarole District, Uganda.

Of the 305 participants, 238 reported having taken an HIV test and received results in the past 12 months, for an HIV testing utilization rate of 78.0% (95% CI: 73.0–82.6%) among the participants. Of the 238 participants who tested for HIV, 208 (87%) voluntarily took the test (Figure 1), while the remaining 30 (13%) were required to take the HIV test.

**Figure 2:**
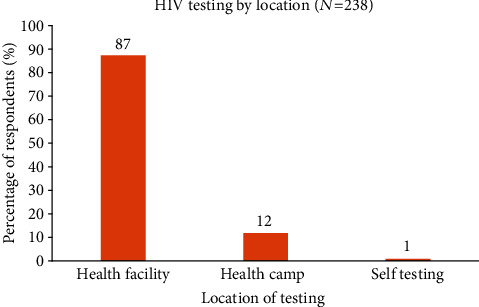
Location for taking an HIV test by boda-boda riders in Fort Portal Municipality, Kabarole District, Uganda.

Among the 238 participants who tested for HIV, 208 (87%) had the test taken at a health facility, 28 (12%) tested during health camps, and the remaining 2 (0.8%) did self-testing, as seen in Figure 2.

**Figure 3:**
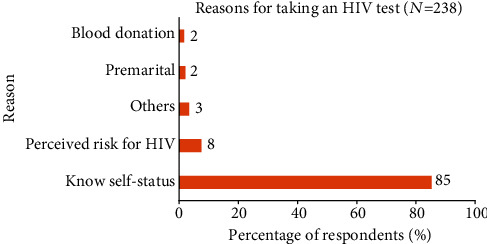
Reasons for taking an HIV test among boda-boda riders, Fort Portal Municipality, Kabarole District, Uganda.

The reasons for taking HIV tests among the 238 participants are presented in Figure 3. The majority of the participants (85%, *n* = 203) wanted to know their status; 18 (7.6%) tested because of perceived risk of HIV; five (2.1%) tested as a premarital requirement; four (1.7%) tested prior to blood donation.

### 3.4. Factors Associated with HIV Testing

#### 3.4.1. Univariable Logistic Regression Analysis

In an unadjusted analysis, participants who were significantly more likely to test for HIV were those who knew the HIV status of their primary partners (OR = 3.40; 95% CI: 1.10–10.52, *P* = 0.034) and those who had good knowledge about HIV/AIDS (OR = 3.02; 95% CI: 1.35–6.77, *P* = 0.007), as shown in Table 3. Participants who were significantly less likely to test for HIV were those in the age category of ≥30 years (OR = 0.35; 95% CI: 0.19–0.66, *P* = 0.001) and those who had spent >10 years in boda-boda business (OR = 0.43; 95% CI: 0.2–0.88, *P* = 0.022).

#### 3.4.2. Multivariable Logistic Regression Analysis

In an adjusted analysis, independent factors associated with the utilization of HIV testing services were age category ≥ 30 years (aOR = 0.33; 95% CI: 0.16–0.70, *P* = 0.004), knowing HIV status of primary partner (aOR = 4.23; 95% CI: 1.24–14.49, *P* = 0.022), having HIV-related stigma (aOR = 0.27; 95% CI: 0.08–0.88, *P* = 0.030), and having good knowledge on HIV/AIDS (aOR = 3.94; 95% CI: 1.65–9.41, *P* = 0.002), as shown in Table 3.

## 4. Discussion

HIV testing is one of the strategies employed globally for prevention and control of the HIV pandemic. This study assessed the HIV testing utilization and identified factors influencing utilization of HIV testing services among boda-boda riders in Fort Portal Municipality, Kabarole District, southwestern Uganda.

The findings from this study indicate that a large proportion (78%) of the boda-boda riders had utilized HIV testing services in the past 12 months. This is higher than the HIV testing coverage of 54% in the general population in the district [16]. A similar study among boda-boda riders in central Uganda reported a lower prevalence of HIV testing of 36.9% than ours. Bwambale and colleagues documented a low testing uptake of 26%, more than a decade ago among men in the neighboring district of Kasese [17]. A study in Western Kenya reported a prevalence of ever testing, of 71.9% among boda-boda operators [18], which is lower than ours, given that we considered those who had tested in the past 12 months. Still, in Coastal Kenya, a lower prevalence of utilization of HIV testing of 53.0% than ours was reported among boda-boda riders. Much lower HIV testing utilization rates across Africa among male populations that range from 3 to 25% have been reported in Ethiopia [19], Malawi [20], and South Africa [21]. The higher uptake of HIV testing among the boda-boda riders compared to their male counterparts may be attributed to the higher perceived risk of contracting HIV, associated with the boda-boda occupation, than the general population [6]. In addition, there has been an expansion of testing services, health education, and rigorous awareness-raising campaigns over the years by the government and nongovernmental organizations [22, 23]. This is undoubtedly the reason for the observed high utilization rate of HIV testing services in the current study, given that most studies under comparison were conducted more than 5–10 years ago. Nevertheless, consistent with the recent demographic and health survey [24], our findings seem to suggest an upward trend in the uptake of HIV testing services.

The study findings showed an association between age and utilization of HIV testing services; riders aged 30 years and above were less likely to take an HIV test. This is consistent with studies conducted in Uganda [17] and elsewhere in African countries [21, 25–28]. This association is due to the fact that older people are less likely to engage in risky sexual behavior and thus have a lower perceived risk of HIV infection compared to the younger individuals. Therefore, deliberate efforts should be made to target older boda-boda riders for HIV testing.

Consistent with previous studies [17, 27], participants who were aware of their primary partner's HIV status were more likely to have an HIV test. Disclosure of HIV status has a positive impact on discrimination and stigma. Therefore, individuals who discuss with their partners about HIV/AIDS are more likely to utilize HIV testing. Our findings underscore the need to encourage open communication and disclosure among partners in order to improve uptake of HIV testing services, as previously suggested [29].

In this study, participants with good knowledge about HIV/AIDS were more likely to utilize HIV testing services. This is in agreement with previous studies [27, 28, 30, 31], which showed that increasing level of knowledge on HIV/AIDS was associated with an increased tendency to utilize HIV testing services. Good knowledge diminishes the misconceptions and myths held by individuals about HIV/AIDS, which are a great barrier to utilization of HIV testing services.

Boda-boda riders with high levels of anticipated HIV-related stigma were less likely to utilize HIV testing services, compared to their counterparts in the current study. Consistent with earlier studies [17, 19, 27, 32–34], our findings further highlight HIV-related stigma as a key hindrance to utilization of HIV testing services. Although HIV-related stigma was low (4.6%) among the study participants, there is a need to design appropriate interventions such as formulating appropriate educational messages to further minimize HIV-related stigma, given its negative effect on utilization of HIV testing services. In addition, there may be need to adopt alternative models of testing such as home-based HIV counseling and testing that diminishes stigma associated with being seen at health facility clinics for voluntary counseling and testing (VCT) [35]. Moreover, a reduction in the levels of discrimination and stigma is a key indicator of the success of programs focusing on HIV/AIDS control and prevention [36].

## 5. Limitations

This study is subject to some limitations. First, we cannot make causal inferences from the observed associations because of the cross-sectional nature of the study design. Second, the findings from this study may be prone to recall and social desirability bias, since information was self-reported and participants were asked to recall information in previous 12 months. Finally, the study was done in a municipality in one district of southwestern Uganda. Therefore, generalization of the findings beyond the study population should be done with caution.

## 6. Conclusion

The utilization of HIV testing services among boda-boda riders in Fort Portal Municipality, Kabarole District, was high. Having good knowledge about HIV/AIDS and knowing the HIV status of the primary partner were associated with increased utilization of HIV testing services. Boda-boda riders with high levels of HIV-related stigma and those who were older (30 years and above) were less likely to utilize HIV testing services. More effort should focus on targeting older boda-boda riders for HIV testing, reduction of HIV-related stigma, improving knowledge on HIV/AIDS, and encouraging communication and disclosure between partners, in order to consolidate the gains made in HIV testing services in this bridge population.

## Figures and Tables

**Table 1:** Demographic characteristics of study participants, by HIV testing utilization status.

Characteristic	Overall (*N* = 305)	Utilized HIV testing in past 12 months		*P* value
		Yes (*N* = 238)	No (*N* = 67)	
Age in years, mean (SD)	32 (±7.1)	31 (±7.3)	34 (±6.2)	0.013
Age category in years, *n* (%)				0.001
18-29	122 (40.0)	107 (45.0)	15 (22.4)	
30 and above	183 (60.0)	131 (55.0)	52 (77.6)	
Level of education, *n* (%)				0.386
None	14 (4.6)	10 (4.2)	4 (6.0)	
Primary	188 (61.6)	142 (59.7)	46 (68.7)	
Secondary	93 (30.5)	77 (32.4)	16 (23.9)	
Tertiary	10 (3.3)	9 (3.8)	1 (1.5)	
Religion, *n* (%)				0.873
Catholic	148 (48.5)	118 (49.6)	30 (44.8)	
Anglican	93 (30.5)	69 (29.0)	24 (35.8)	
Moslem	35 (11.5)	28 (11.8)	7 (10.5)	
Pentecostal	19 (6.2)	15 (6.3)	4 (6.0)	
Others	10 (3.0)	8 (3.4)	2 (3.0)	
Weekly income^§^, *n* (%)				0.304
<50,000	71 (23.3)	51 (21.4)	20 (29.9)	
50,000-100,000	214 (70.2)	172 (72.3)	42 (62.7)	
>100,000	20 (6.6)	15 (6.3)	5 (7.5)	
Duration in occupation in years, *n* (%)				0.039
<5 years	94 (30.8)	78 (32.8)	16 (23.9)	
5-10 years	137 (44.9)	110 (46.2)	27 (40.3)	
>10 years	74 (24.3)	50 (21.0)	24 (35.8)	
Marital status, *n* (%)				0.853
Married, living with partner	238 (78.0)	186 (78.2)	52 (77.6)	
Married, not living with partner	19 (6.2)	13 (5.5)	6 (9.0)	
Divorced/separated	10 (3.3)	9 (3.8)	1 (1.5)	
Not married, living with partner	10 (3.3)	8 (3.4)	2 (3.0)	
Widowed	2 (0.7)	2 (0.8)	0 (0.0)	
Not married, not living with partner	26 (8.5)	20 (8.4)	6 (9.0)	
Number of wives, *n* (%)				0.744
One wife	188 (61.6)	144 (60.5)	44 (65.7)	
More than one wife	71 (23.3)	57 (24.0)	14 (20.9)	
Not applicable	46 (15.1)	37 (15.6)	9 (13.4)	
Have children, *n* (%)				0.215
No	29 (9.5)	20 (8.4)	9 (13.4)	
Yes	276 (90.5)	218 (91.6)	58 (86.6)	

^§^Weekly income in Ugandan shillings.

**Table 2:** Health, behavioral, and social characteristics of study participants, by HIV utilization.

Characteristic	Overall (*N* = 305)	Utilized HIV testing in the past 12 months		*P* value
		Yes (*N* = 238)	No (*N* = 67)	
Ever visited health facility, *n* (%)				0.392
No	2 (0.7)	1 (0.4)	1 (1.5)	
Yes	303 (99.3)	237 (99.6)	66 (98.5)	
Reason for visiting health facility, *n* (%)				<0.001
To see a sick relative	56 (18.4)	37 (15.6)	19 (28.4)	
To deliver commodity	14 (4.6)	9 (3.8)	5 (7.5)	
When I was sick	66 (21.6)	45 (18.9)	21 (31.3)	
Other reasons	157 (51.5)	141 (59.2)	16 (23.9)	
Not applicable	2 (0.7)	0 (0.0)	2 (3.0)	
Person recently had sex with, *n* (%)				0.386
Not primary partner	36 (11.8)	30 (12.6)	6 (9.0)	
Primary partner	263 (86.2)	207 (87.0)	57 (85.1)	
Refused to answer	2 (0.7)	1 (0.4)	1 (1.5)	
Not applicable	4 (1.3)	3 (1.3)	1 (1.5)	
Knows HIV status of primary partner, *n* (%)				0.050
No	13 (4.3)	7 (2.9)	6 (9.0)	
Yes	273 (89.5)	218 (91.6)	55 (82.1)	
Not applicable	19 (6.2)	13 (5.5)	6 (9.0)	
History of smoking, *n* (%)				0.296
No	286 (93.8)	225 (94.5)	61 (91.0)	
Yes	19 (6.2)	13 (5.5)	6 (9.0)	
Currently smoking, *n* (%)	18 (5.9)	12 (5.0)	6 (9.0)	1.000
Alcohol consumption, *n* (%)				0.238
No	174 (57.1)	140 (58.8)	34 (50.8)	
Yes	131 (43.0)	98 (41.2)	33 (49.3)	
Perceived risk of HIV infection in coming year, *n* (%)				0.533
High	25 (8.2)	18 (7.6)	7 (10.5)	
Moderate	47 (15.4)	34 (14.3)	13 (19.4)	
Low	117 (38.4)	92 (38.7)	25 (37.3)	
No risk at all	111 (36.4)	89 (37.4)	22 (32.8)	
Do not know	5 (1.6)	5 (2.1)	0 (0.0)	
HIV-related stigma, *n* (%)				0.053
No	291 (95.4)	230 (96.6)	61 (91.0)	
Yes	14 (4.6)	8 (3.34)	6 (9.0)	
Knowledge on HIV/AIDS, *n* (%)				0.005
Poor	28 (9.2)	16 (6.7)	12 (17.9)	
Good	277 (90.8)	222 (93.3)	55 (82.1)	

**Table 3:** Univariable and multivariable logistic regression analyses for factors associated with HIV testing among boda-boda riders in Fort Portal Minicipality, southwestern Uganda.

Characteristic	% tested for HIV	Univariable analysis		Multivariable analysis	
	*n*/*N* (%)	Crude OR (95% CI)	*P* value	Adjusted OR (95% CI)	*P* value
Age category					
18-29	107/122 (87.7)	Ref		Ref	
30 and above	131/183 (71.6)	0.35 (0.19-0.66)	0.001	0.33 (0.16-0.70)	0.004
Duration in boda-boda business					
<5 years	78/94 (83.0)	Ref		Ref	
5-10 years	110/137 (80.3)	0.84 (0.42-1.65)	0.607	0.89 (0.42-1.93)	0.776
>10 years	50/97 (67.6)	0.43 (0.21-0.88)	0.022	0.69 (0.29-1.64)	0.399
Perceived risk of HIV infection in coming year					
High	18/25 (72.0)	Ref		Ref	
Moderate	34/47 (72.3)	1.02 (0.34-3.00)	0.976	1.40 (0.44-4.48)	0.571
Low	92/117 (78.6)	1.43 (0.54-3.81)	0.473	1.53 (0.54-4.39)	0.424
No risk at all	89/111 (80.2)	1.57 (0.58-4.23)	0.37	1.82 (0.62-5.33)	0.273
Do not know	5/5 (100)	N/A	N/A	N/A	N/A
Knows HIV status of primary partner					
No	7/13 (53.8)	Ref		Ref	
Yes	218/273 (79.9)	3.40 (1.10-10.52)	0.034	4.23 (1.24-14.49)	0.022
HIV-related stigma					
No	230/291 (79.0)	Ref			
Yes	8/14 (57.1)	0.35 (0.12-1.06)	0.063	0.27 (0.08-0.88)	0.030
Knowledge on HIV/AIDS					
Poor	16/28 (57.1)	Ref		Ref	
Good	222/277 (80.1)	3.02 (1.35-6.77)	0.007	3.94 (1.65-9.41)	0.002

Ref: reference category; OR: odds ratio; CI: confidence interval; N/A: not applicable.

## Data Availability

The datasets generated and analyzed during the study are available from the corresponding author on request.
